# Integrated Analysis of Gene Network in Childhood Leukemia from Microarray and Pathway Databases

**DOI:** 10.1155/2014/278748

**Published:** 2014-04-15

**Authors:** Amphun Chaiboonchoe, Sandhya Samarasinghe, Don Kulasiri, Kourosh Salehi-Ashtiani

**Affiliations:** ^1^Centre for Advanced Computational Solutions (CfACS), Lincoln University, Lincoln 7647, New Zealand; ^2^Division of Science and Math, New York University Abu Dhabi and Center for Genomics and Systems Biology (CGSB), New York University Abu Dhabi Institute, P.O. Box 129188, Abu Dhabi, UAE; ^3^Integrated Systems Modelling Group, Lincoln University, Lincoln 7647, New Zealand; ^4^Department of Wine, Food & Molecular Biosciences, Lincoln University, Lincoln 7647, New Zealand

## Abstract

Glucocorticoids (GCs) have been used as therapeutic agents for children with acute lymphoblastic leukaemia (ALL) for over 50 years. However, much remains to be understood about the molecular mechanism of GCs actions in ALL subtypes. In this study, we delineate differential responses of ALL subtypes, B- and T-ALL, to GCs treatment at systems level by identifying the differences among biological processes, molecular pathways, and interaction networks that emerge from the action of GCs through the use of a selected number of available bioinformatics methods and tools. We provide biological insight into GC-regulated genes, their related functions, and their networks specific to the ALL subtypes. We show that differentially expressed GC-regulated genes participate in distinct underlying biological processes affected by GCs in B-ALL and T-ALL with little to no overlap. These findings provide the opportunity towards identifying new therapeutic targets.

## 1. Introduction 

Childhood leukaemia, specifically, acute lymphoblastic leukaemia (ALL) can be divided into two subgroups: T-lineage and B-lineage. Glucocorticoids (GCs) are a type of steroid hormones. Synthetic glucocorticoids such as dexamethasone (Dex) and prednisolone (PRD) are the most important drugs that have been used extensively in the treatment of children with acute lymphoblastic leukaemia (ALL) because of their ability to induce apoptosis (cell death) in the lymphoid. There are more than 2000 studies on GC-induced apoptosis in lymphoid cells [1], of which some studies focus on identifying GC-regulated genes by using gene expression profiles from different types of glucocorticoid drugs in different clinical settings (*in vivo*,* in vitro*, and human samples) [2–4]; however, there are only a few overlapping genes identified from these studies. Therefore, there is a need to verify GC-regulated genes to better define the underlying network of genes involved in the actions of GCs.

In this study, we illustrated how a combination of existing bioinformatics tools can address the heterogeneity of ALL in three different levels: gene, gene set, and network and pathway. First, at the gene level, we identified GCs-regulated candidate genes for each subtype. The second level was to use a group of genes (gene sets) that may have similar functions. We aim to ascertain whether each gene set from each subtype is significantly enriched in a list of selected phenotypes. The third level was to construct gene networks of the proposed GCs regulated genes from three selected web-based tools: Ingenuity Pathway Analysis (IPA), Search Tool for The Retrieval of Interacting Genes (STRING), and Gene Multiple Association Network Integration Algorithm (GeneMANIA).

## 2. Materials and Methods

### 2.1. Dataset

**Figure 1:**
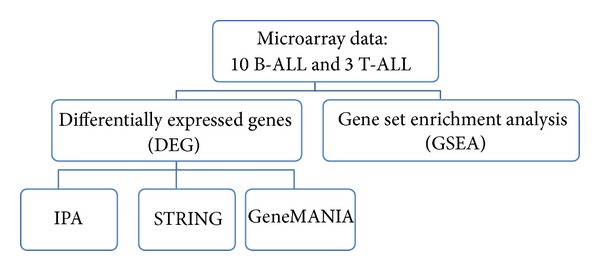
Summary of dataset analysis methodology used in this study.

Raw microarray data in the format of CEL files were obtained online from the Gene Expression Omnibus (GEO) (http://www.ncbi.nlm.nih.gov/geo/). Raw data, comprising gene expression measurements for 13 patients (three T-ALL patients and ten B-ALL patients), contained gene expression measurements collected at three time points: 0 hour, 6/8 hours, and 24 hours. The experiments and analysis conducted on the data are described in Schmidt et al. [5]. Summary of dataset analysis methodology used in this study is shown in Figure 1.

### 2.2. Normalization

The normalization used in this study is Robust Multiarray Average (RMA) [6]. RMA uses a global correction, quantile normalization, and median polish summarization. There are many existing software and tools for RMA calculation available from both commercial and free sources. This study used R (http://www.r-project.org/) and BioConductor (http://www.bioconductor.org/). The differentially expressed genes were selected from the normalised data according to specified threshold activation (log-ratio of ±1 or higher or ±0.7 or higher).

### 2.3. Gene Set Enrichment Analysis and Enrichment Map

Gene set enrichment analyses (GSEA) [7, 8] are commonly used to determine the biological characterization, statistical significance, and concordant differences between an experimental gene set and a selected gene list from annotated gene sets knowledge base stored on Molecular Signatures Database (MSigDB). GSEA can be downloaded from http://www.broadinstitute.org/gsea/downloads.jsp. The Jaccard coefficient is used to compare the similarity between two sample gene sets A and B and defined as the intersection between group A and B divided by their union. The results from GSEA are then visualized through the enrichment map [9], a Cytoscape plugin for network visualization. The ranked experimental gene list along with the enriched gene sets from GSEA is used to build the network of gene sets (nodes) where edges represent their similarity. Size of a node varies by gene set size and the thickness of the edge represents the degree of correlation between two gene sets.

### 2.4. Networks and Pathway Analysis

#### 2.4.1. Ingenuity Pathway Analysis Software (IPA)

IPA (Ingenuity Systems, Redwood City, CA, USA, http://www.ingenuity.com/) is a web-based application that applies a systems biology approach to solve various biological problems. The knowledge base of IPA has been curated from journal articles, textbooks, and other data sources. This software has many applications; only functional analysis of genes and their networks were used in this study. The *p* value defines the significance of gene function in a network as well as gene to gene relation, and a *p* value less than 0.05 signifies a statistically significant and nonrandom association. The right-tailed Fisher Exact Test is used to calculate *p* value.

#### 2.4.2. Analysis of Network Invoked by GC-Regulated Genes

The biological knowledge of gene and protein interactions is growing rapidly and there are many tools and curated databases available on a large scale. Insightful knowledge gained from studying gene sets rather than individual genes using network-based approaches can reveal network patterns and relevant molecular pathways from the experiment gene sets. In this study, we utilized two different freely accessible and user-friendly web tools as follows.

Gene Multiple Association Network Integration Algorithm (GeneMANIA) [10, 11] (http://www.genemania.org/) is a web-based tool for prediction of gene function or implemented as a Cytoscape plugin tool. Based on single gene or gene set query from 7 organisms, it shows results for interactive functional associative network according to their coexpression data from Gene Expression Omnibus (GEO), physical and genetic interaction data derived from BioGRID, predicted protein interaction data based on orthology from I2D, colocalization, shared protein domain, and GO function.

Search Tool for the Retrieval of Interacting Genes (STRING) version 9.1 [12, 13] (http://string-db.org/) is an online protein-protein interaction database curated from literature and predicted associations from systemic genome comparisons. User can query using single or multiple name(s) and protein sequence(s). The protein interactions can be displayed according to their confidence, evidence, actions, or interactions.

## 3. Results and Discussion

### 3.1. Identification of GC-Regulated Genes through a Refined Analysis of Data

Our initial analysis of this time series gene expression data for differentially expressed gene identification followed the same method used by the original authors [5]. All 39 files were processed and normalised by Robust Multiarray Average (RMA) in R as in the original study. The selected normalization method may have an effect on downstream analysis, for example, reverse engineering analyses [14]; however, investigating this effect is beyond the scope of this study. We found that combining B-ALL and T-ALL data (as done by the original authors) compromises the accuracy of selection of differentially expressed. For example, the original authors found 22 differentially expressed genes from the combined dataset. However, a closer inspection revealed to us that only 8/22 candidate genes belonged to both B-ALL and T-ALL and 14/22 genes were found only in B-ALL or T-ALL.

Therefore, we separated the data from the two types of patients and a new set of differentially expressed genes was selected for T-ALL and B-ALL for each time point. Our new criteria used were log ratio of ±1 or higher for at least five out of ten (50%) B-ALL patients and two out of three T-ALL patients. We also analysed data for early response (6 hours) and late response (24 hours), but we added an analysis of response between 6 and 24 hours because this can give more information about gene activity at different periods. The results are shown in Table 1.

Table 1 shows the number of differentially expressed genes 6 hours after treatment, between 6 hours and 24 hours, and 24 hours after treatment (before and after deleting cell cycle genes) with our new criteria. Before deleting the cell cycle genes, the final set had 237 probe sets (203 unique probe sets after removing repeats) for T-ALL for the combined time points and 257 probe sets (207 unique probe sets) for B-ALL and these were combined into one set. The final set contained 386 unique probe sets (24 unique probe sets were common to T-ALL and B-ALL, of which three probes were not found by the original authors. These were converted from probe set ID to gene symbol by using DAVID (the Database for Annotation, Visualization and Integrated Discovery). Then the cell cycle genes were deleted from this dataset (cell cycle gene list was retrieved from KEGG, Cell cycle database, and the original article).

After deleting cell cycle genes, T-ALL contained 222 probe sets (172 unique probe sets) and B-ALL contained 190 probe sets (155 unique probe sets) for the combined time points. The final set had 308 unique probe sets (304 genes) responsive to GCs (19 unique probe sets were common to T-ALL and B-ALL).

We then compared our new gene list with the genes reported in previous studies. None of the previous studies using the same drugs and the same times at which data were collected show similar results. Our main focus here is to use relevant experimental data from clinical samples, such as the study by Tissing et al. [15], where the investigators used the same drug on retrieved tissues from childhood leukemia patients, not cell cultures, but used a different normalization process, variance stabilization procedure (VSN), than of our study. They compared the primary childhood ALL cells treated with* in vivo* prednisone and leukemic cells of childhood ALL exposed to* in vitro* prednisolone, for finding early apoptosis responsive genes at 8 hours after treatment. Our 29 differentially expressed probe sets from T-ALL and 47 probe sets from B-ALL (Table 1, first two columns containing cell cycle genes) were compared with the 39 upregulated and 12 downregulated genes from Tissing et al. [15]. We found four common induced genes: BTG1 (T-ALL), FKBP5, ZBTB16, and SNF1LK (B-ALL). However, no common repressed genes were found between our results and Tissing et al. [15] for either B-ALL or T-ALL.

We selected another study, Thompson and Johnson [16], based on different chemotherapeutic drugs, different time points, and different tissues, but same gene selection criteria as ours. Thompson and Johnson [16] identified 39 upregulated genes and 21 downregulated genes in CEM (a cell line derived from human lymphoid cells). In addition, they proposed a time frame for apoptosis gene regulation after CEM-C7 were exposed to dexamethasone [16]. Comparing gene sets reported by Thompson and Johnson [16] with our differentially expressed genes from T-ALL and B-ALL patients, we found few overlapping genes, but more than what we found from comparison with Tissing et al. [15]. T-ALL had five overlapping genes (BCL2L1, SOCS1, BTG1, CD69, and NR3C1) and B-ALL had four overlapping genes (SOCS1, DFNA5, WFS1, and SLA). Of these two sets, BTG1 is the only common gene between our T-ALL, Tissing et al. [15] and Thompson and Johnson [16]. There were no common genes between our B-ALL, Tissing et al. [15] and Thompson and Johnson [16].

### 3.2. Extraction of Intrinsic Biological Patterns with Gene Enrichment Analysis Applied to Gene Expression Data

Identification of differentially expressed genes between the two classes has limited value in gaining biological insights unless it is integrated with other analyses. The gene set enrichment analysis (GSEA) method paves the way to interpret gene expression data by using prior knowledge databases to define functionally characterized gene sets and to reveal whether the identified gene sets have common biological functions or gene ontologies.

**Figure 2:**
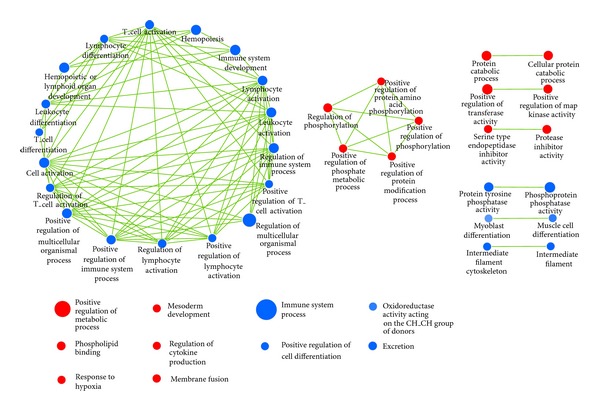
Gene set enrichment analysis delineates gene ontology (GO) that differentiates between B- and T-ALL with respect to biological processes. Gene set enrichment analysis (GSEA) comparing B-ALL (red) and T-ALL (blue) in ALL dataset, illustrating differentiation of gene ontology (biological processes) between two subgroups (5% FDR, *p* = 0.05). Cytoscape and enrichment map were used for visualization of the GSEA results; only gene sets from MSigDB C5 (gene ontology) were used. Nodes represent enriched GO gene sets, whose size reflects the total number of genes in that gene set. Edge thickness (green line) represents the number of overlapping genes between gene sets calculated using Jaccard coefficient. Single nodes and 2-node interactions for both B- and T-ALL, a 5 node-interaction for B-ALL, and interaction between a large number of nodes for T-ALL are shown.

ALL gene expression data corresponding to all 54,675 probe sets on the chip were collapsed into 20,606 gene symbols. A gene set S, a subset derived from the gene symbols, was used to calculate the enrichment score, which placed the set S, according to statistical significance, at the top or bottom of the selected list L of gene sets from MSigDB (Molecular Signature Database, version 4.0 updated May 31, 2013). The list L that was used in this study consisted of two types: (1) functional set (C_2_) 4722 gene sets collected from KEGG online pathway database and (2) GO gene set (C_5_) 1454 gene sets according to the GO terms. The differentially expressed genes found in this study belonged to 176 gene sets and out of these 130 gene sets were upregulated in B-ALL and 46 gene sets were upregulated in T-ALL. For B- and T-ALL, 12 and 8 gene sets were significantly enriched at nominal *p* value <5%, respectively.  Table 2 shows the different KEGG pathways where B- and T-ALL gene sets were enriched. The limitation of GSEA is the gene set redundancy and difficulty in interpreting the results from large gene lists. We used the enrichment map, a Cytoscape plugin, to build the network from GSEA Go term enrichment results as shown in Figure 2. Node size defines the number of genes in each gene set and edge thickness represents the proportion of overlap between gene sets, calculated using the Jaccard coefficient. Blue represents T-ALL gene sets and red represents B-ALL gene sets. From Table 2 and Figure 2, we differentiate the functional characteristics of metabolic pathways (KEGG) and biological processes (GO) in which B- and T-ALL are involved. These distinctions are as follows: B-ALL is likely to be involved in asthma, B-cell receptor signalling pathway, and phosphorylation, while T-ALL is involved in T-cell receptor signalling pathway, primary immunodeficiency process, and leucocyte.

### 3.3. Networks and Pathways Analysis

#### 3.3.1. Inferring GR Gene Networks from GC-Induced Apoptosis Genes

Dataset containing expression values of GCs-regulated genes were uploaded and analyzed using Ingenuity Pathways Analysis software (Ingenuity Systems, http://www.ingenuity.com/). Ingenuity Pathway Analysis software (IPA) maps the genes to pathways generating networks using an algorithm based on gene connectivity with cutoff of 35 molecules per network.

We used the gene list after deleting cell cycle genes to infer gene networks through IPA software. For T-ALL, there are 28 probe sets at 0–6 hours, 105 probe sets at 6–24 hours and 89 probe sets at 0–24 hours. For B-ALL there are 33 probe sets at 0–6 hours, 25 probe sets at 6–24 hours, and 132 probe sets at 0–24 hours. Results from IPA typically are a number of networks ranking from one to seven for each time point with a maximum of 35 molecules in each network consisting of those genes from our list plus those added by IPA. Processing of these networks were fairly time consuming, but we manually identified common genes active throughout the whole period (at least between two time points) which can be referred to as genes predominant in T- and B-ALL separately or common to both.

Overall, for T-ALL patients, 48 unique genes (23 genes were given as input and 25 were added by IPA) were found for the three different time points from IPA. For B-ALL patients, 47 unique genes (21 genes were given as input and 26 were added by IPA) were found.

In the next step, we only selected genes that were found at least in two of the three time intervals from our list to create gene networks. T- and B-ALL patients were quite different in the “molecular and cellular functions” and “canonical pathways and functions.” For example, molecular and cellular functions of T-ALL were involved more in cell death, while those of B-ALL were more involved in cell cycle. This finding may imply that (i) apoptosis process in T-ALL may occur before B-ALL during the same period of treatment; (ii) there are many cells progressing through cell cycle (cycling cells) in B-ALL, while many noncycling cells are in T-ALL. Many pathways/steps are involved in cycling cells than in non-cycling cells in the apoptosis process after glucocorticoid treatment [17]. Genes found in both T-and B-ALL were involved in cancers functions.

#### 3.3.2. Analysis of Network Invoked by GC-Regulated Genes

Network analysis can help understand the molecular and cellular interactions [18]. It can be visualized to represent entities (nodes) and their relationships (arcs). The advent of high throughput technology has led to a large increase in publicly available information. Each data type can capture different aspect of functional roles of interested genes. In this section, we investigated functional interaction among genes and proteins in the cell using available data and knowledge bases. We selected two different web-based network tools: GeneMANIA and STRING using the differentially expressed genes from early response of B- and T-ALL as a query gene sets. These toolsets integrates computational methods to predict the gene functions based on a collection of interaction networks.

GeneMANIA is a large collection of interaction networks from several data sources which identify genes and networks that are functionally associated (protein and genetic interactions, pathways, coexpression, colocalization, and protein domain similarity) with the query gene sets. Another advantage is that the user can run this as a plugin with the Cytoscape tool allowing the user to apply other tools to analyze the networks. STRING relies on the phylogeny to infer the functional interaction (protein networks) with direct interaction to score nodes, while GeneMANIA uses functional genomic data with label propagation to score nodes and generate gene networks. STRING uses precomputed networks, while those of GeneMANIA are not precomputed, and user can upload their own networks. STRING covers a large number of organisms, while GeneMANIA only covers 7 organisms but allows the user to upload or add more networks through the plugin. In addition, users can run enrichment analysis (GO, KEGG, PFAM, INTERPRO, and protein-protein interaction) on STRING.

**Figure 3:**
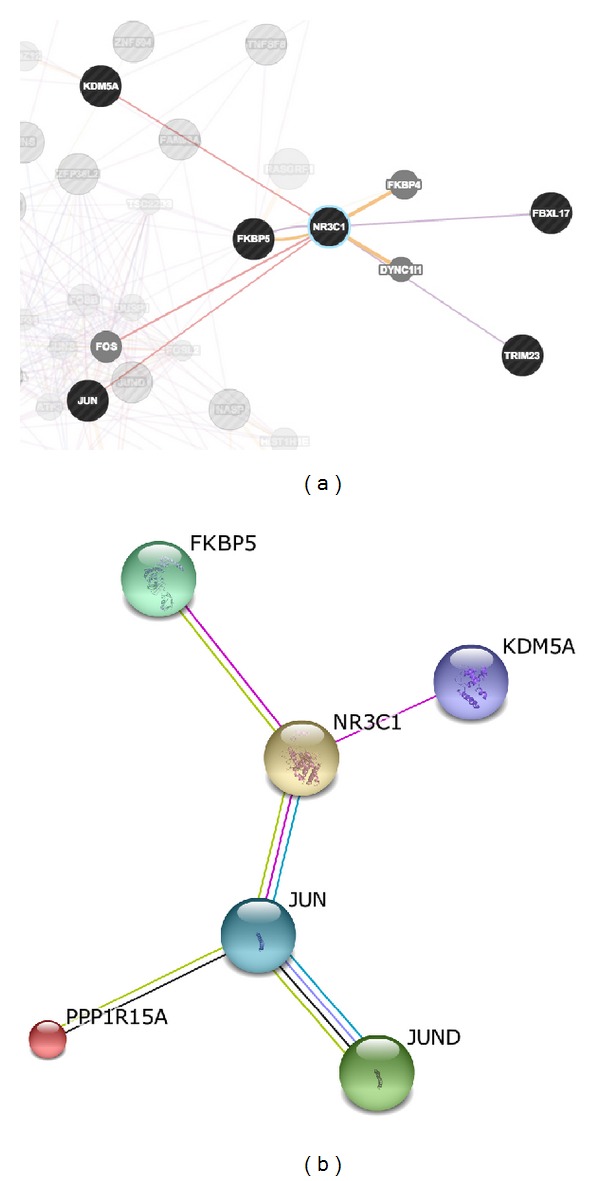
Gene network of T-ALL (early response) derived from GeneMANIA (a) and STRING (b) (NR3C1 interaction is highlighted). (a) A gene network from GeneMANIA shows the relationships for genes from the list (nodes) connected (with edges) according to the functional association networks from the databases. (b) The figure illustrates the protein interaction upon querying STRING protein network (evidence view) in* Homo sapiens* with 49 proteins. Additional information from other resources can be retrieved for each protein and interaction. Nodes represent proteins and different line colours denote the type of evidence for the interaction.

The results of genes and network that are functionally associated with the gene set from early response of T-ALL are shown in Figure 3. We compared the two networks from STRING and GeneMANIA based on the interactions they revealed; here we used NR3C1 as the centre gene in the comparison. All interactions found in STRING were found in GeneMANIA as described in more detail in Table 3. Comparison between the results from both tools can be used to confirm the functional associations of the interested gene sets. There is evidence of overlap and uniqueness in the interactions revealed by the two web-based tools.

## 4. Summary and Conclusions

We used a dataset from Schmidt et al. [5], the most prominent dataset at the time because it used gene expression data collected from childhood leukemia patients at two time points after treatment. We found that the selected dataset is reproducible and robust. The original differentially expressed genes were proposed by the authors for the combined dataset; however, only some of these genes were found in each subtype. To resolve the discrepancy, we proposed a new criteria for the two subtypes separately (log 2 ratio of ±1.0 for five out of ten B-ALL patients and two out of three T-ALL patients) and new gene sets were generated. Furthermore, we extended the analysis to find differentially expressed genes between 6 and 24 hours. In addition, we compared this gene set with differentially expressed gene sets from two previous studies and found only a few common genes possibly indicating that different chemotherapeutic agents and tissues may produce different results for the target gene set.

We identified common genes by manually extracting connections from inferred gene networks for each time interval. In addition, results from IPA showed different molecular and cellular functions, canonical pathways and functions between T- and B-ALL. T-ALL is more involved in cell death, while B-ALL is more involved in cell cycle.

Converting gene list to gene sets, we identified the known metabolic pathways that were enriched in each subtype using GSEA with enrichment map. We subsequently compared the top 5 gene ontology (GO) functions for B- and T-ALL. Then two network based tools: GeneMANIA and STRING were used to identify the gene network. STRING uses protein names to search for known and predicted protein interactions while GeneMANIA uses gene symbols to search for gene function prediction(s). Comparing gene interaction for T-ALL from GeneMANIA and STRING, we found some overlap.

In summary, we utilized the strengths of existing network/pathway tools and databases to gain insight into processes related to childhood leukemia subtypes; T-ALL and B-ALL have distinct molecular interaction patterns visible from various systems levels, including gene, gene set, molecular pathway, and gene networks. Discriminating between the two groups can help to improve the understanding of a drug's mechanism and further improve targeting in therapeutics drug research. In addition, the future research should consider combining RNA-Seq data [19–21] for identification of novel prognostic markers and therapeutic targets.

## Figures and Tables

**Table 1:** Differentially expressed probe sets 6 hours after treatment, between 6 hours and 24 hours, and 24 hours after treatment at ±1 log2 ratio fold change (before and after deleting cell cycle genes).

	0–6 hours				6–24 hours				0–24 hours			
	Before		After		Before		After		Before		After	
	Induced	Repressed	Induced	Repressed	Induced	Repressed	Induced	Repressed	Induced	Repressed	Induced	Repressed
T-ALL	19	10	19	9	59	51	56	49	58	40	56	33
B-ALL	24	23	24	9	16	13	16	9	73	108	71	61

**Table 2:** Top 5 of KEGG enrichment term of B- and T-ALL.

Enriched in B-ALL	FDR	Enriched in T-ALL	FDR
KEGG_ASTHMA	0.001	KEGG_T_CELL_RECEPTOR_SIGNALING_PATHWAY	0.0003
KEGG_B_CELL_RECEPTOR_SIGNALING_PATHWAY	0.002	KEGG_PRIMARY_IMMUNODEFICIENCY	0.07
KEGG_ANTIGEN_PROCESSING_AND_ PRESENTATIONKEGG_LEISHMANIA_INFECTION	0.003	KEGG_HEMATOPOIETIC_CELL_LINEAGE	0.209
KEGG_LEISHMANIA_INFECTION	0.009	KEGG_BIOSYNTHESIS_OF_UNSATURATED_FATTY_ACIDS	0.162
KEGG_TYPE_I_DIABETES_MELLITUS	0.058	KEGG_ALPHA_LINOLENIC_ACID_METABOLISM	0.210

**Table 3:** Interactions for T-ALL (early response) network using GeneMANIA and STRING.

Interaction	GeneMANIA	STRING
NR3C1 → FKBP5	Coexpression Predicted	Coexpression Comentioned in PubMed abstracts
NR3C1 → JUN	Physical interaction	Comentioned in PubMed abstracts Association in curated databases, experiments data
NR3C1 → KDM5A	Physical interaction	Experiments data
NR3C1 → STS	Colocalization	—
NR3C1 → TRIM23	Coexpression	—
NR3C1 → EPM2AIP1	Coexpression	—
NR3C1 → CHST11	Coexpression	—
NR3C1 → FBXL17	Coexpression	—
FOS → NR3C1	Physical interaction	—
FKBP4 → NR3C1	Predicted	—
DYNC1l1 → NR3C1	Predicted	—
JUN → JUND	Physical interaction Coexpression Shared protein domains	Coexpression Comentioned in PubMed abstracts Association in curated databases Posttranslational modification
JUN → PPP1R15A	Coexpression Comentioned in PubMed abstracts	Coexpression Comentioned in PubMed abstracts
